# Current Hospital Topics

**Published:** 1906-10-06

**Authors:** 


### HOSPITAL ADMINISTRATION.

### CONSTRUCTION AND ECONOMICS.

### CURRENT HOSPITAL TOPICS.

#### Women and Hospital Management.

If women are to take an active part in the administration of hospitals their services should prove most useful and welcome on the committee of an institution for women. The ladies have always taken a paramount part in the administration of the affairs of the Women's Hospital in Melbourne. Its committee consists of twenty-two members, of whom sixteen are women and six are men. A recent proposal to increase the number of men has been defeated at the annual meeting by an overwhelming majority. We are glad to record this evidence of the efficiency of women workers in one section of hospital administration in Australia.

#### The Hospital Saturday Fund.

Mr. W. G. Bunn, who has been secretary to this Fund for seventeen years, has resigned his position. We regret that his resignation is due to ill-health, for Mr. Bunn was one of the most courteous, industrious and useful men who have been associated with the Hospital Saturday movement from its inception to the present time. Mr. W. G. Bunn has rendered excellent service to the Fund in many directions, and we sincerely regret the loss of his services which have had an especial value and are so likely to be remembered long after his retirement. We hope that the rest may speedily restore Mr. Bunn to his usual good health, and that he will accept the expression of our warm sympathy with him in the circumstances. At the meeting to-day Mr. G. H. Hamilton will propose that the board of delegates appoint Mr. Arthur W. Davies, the present chief organising secretary of the Fund, as Mr. Bunn's successor, at a salary of £250 per annum. Mr. W. H. Reid has been elected assistant organising secretary at a salary of £140 per annum.

#### The Metropolitan Hospital.

This hospital has been singularly blessed by the zealous services of individual workers. The late chairman and founder of the hospital, Mr. Joseph Fry, devoted sixty years of his life to the raising of the necessary income for its work. He was a well-known and popular figure in the city of London, and was always welcome in the counting-house of every large firm. Another invaluable worker, Mr. D. H. Goodsall, F.R.C.S.(Eng.), its senior surgeon, has just died. Mr. Goodsall had identified himself with the management of the hospital, was a member of every committee, and was noted for his regular attendance and useful services. Mr. Goodsall worked for the hospital for thirty-four years, and at the time of his death he was putting forth all his strength to secure a site for the new nurses' home. Mr. Joseph Fry and Mr. Goodsall represent the type of hospital workers which have made the voluntary system of hospital support the wonder and envy of foreign nations.

#### Perth Infirmary.

Dr. Mackintosh's report on the working of this infirmary is likely to be followed by important reforms. On the recommendation of the special committee to which the report was referred it has been agreed by the directors to adopt the uniform system of accounts, a greatly improved system of training and remuneration for probationers, and to discontinue the practice of admitting cases of infectious disease which will now be treated in the new isolation hospital at Friarton. Another important change will be that all requisitions for surgical instruments and expensive drugs must bear the signature of the surgeon or physician requiring them, and be submitted to the house committee. It is probable that Dr. Mackintosh's report may lead to an alteration of the constitution to provide that all public works subscribing £20 annually or upwards may nominate two directors to the board of management. It is admitted the time is approaching when the infirmary must be rebuilt, and the directors are considering the desirability of finding a site outside the burgh in the hope that some generous donor may provide the means of acquiring it.

#### British Seamen and Foreign Hospitals.

It is the glory of the *Dreadnought* at Greenwich that it is free to seamen of all nations, regardless of race, creed, or colour. Any sailor who is sick or the subject of an accident receives free treatment on application. British sailors in foreign ports suffer greatly from the fact that the seamen's hospitals there almost invariably require payment from all foreign sailors who may be admitted as patients. Many of these foreign hospitals are well equipped, but, like that at Hamburg, they refuse to admit sailors except on payment. Mispractice often causes much suffering, and may even have ministered to the death of British sailors. This is a matter which demands the attention of the British authorities at home and abroad. The *Dreadnought* Seamen's Hospital might render very useful service to British sailors by preparing a return of seamen's hospitals in the chief foreign ports most used by British ships and then promoting a movement in favour of the endowment of a certain number of beds in each of these hospitals for the exclusive use of English sailors. The matter is one of very real importance, and we hope this suggestion may be taken up energetically, as the whole sum involved is not great, though the question is one of pressing importance to the British Mercantile Marine Service all the world over.

